# Covalently Immobilized Regenerable Immunoaffinity Layer with Orientation-Controlled Antibodies Based on Z-Domain Autodisplay

**DOI:** 10.3390/ijms23010459

**Published:** 2021-12-31

**Authors:** Jong-Min Park, Mi Yeon Kim, Joachim Jose, Min Park

**Affiliations:** 1 Major in Materials Science and Engineering, Hallym University, Chuncheon 24252, Korea; jongminpark@hallym.ac.kr (J.-M.P.); kimmy_98@naver.com (M.Y.K.); 2 Cooperative Course of Nano-Medical Device Engineering, Hallym University, Chuncheon 24252, Korea; 3 Integrative Materials Research Institute, Hallym University, Chuncheon 24252, Korea; 4 Institute of Pharmaceutical and Medical Chemistry, Westfälische Wilhelms-Universität, 48149 Münster, Germany; joachim.jose@uni-muenster.de

**Keywords:** autodisplay, Z-domains, crosslinker, immunoaffinity layer, covalent immobilization, orientation control, regeneration, SPR biosensor

## Abstract

A regenerable immunoaffinity layer comprising covalently immobilized orientation-controlled antibodies was developed for use in a surface plasmon resonance (SPR) biosensor. For antibody orientation control, antibody-binding Z-domain-autodisplaying *Escherichia coli* (*E. coli*) cells and their outer membrane (OM) were utilized, and a disuccinimidyl crosslinker was employed for covalent antibody binding. To fabricate the regenerable immunoaffinity layer, capture antibodies were bound to autodisplayed Z-domains, and then treated with the crosslinker for chemical fixation to the Z-domains. Various crosslinkers, namely disuccinimidyl glutarate (DSG), disuccinimidyl suberate (DSS) and poly (ethylene glycol)-ylated bis (sulfosuccinimidyl)suberate (BS(PEG)_5_), were evaluated, and DSS at a concentration of 500 μM was confirmed to be optimal. The *E. coli*-cell-based regenerable HRP immunoassay was evaluated employing three sequential HRP treatment and regeneration steps. Then, the Oms of *E. coli* cells were isolated and layered on a microplate and regenerable OM-based HRP immunoassaying was evaluated. Five HRP immunoassays with four regeneration steps were found to be feasible. This regenerable, covalently immobilized, orientation-controlled OM-based immunoaffinity layer was applied to an SPR biosensor, which was capable of quantifying C-reactive protein (CRP). Five regeneration cycles were repeated using the demonstrated immunoaffinity layer with a signal difference of <10%.

## 1. Introduction

The immunoaffinity layer, a molecular recognition layer in immunosensors, is a major component of biosensors, and is generally composed of immobilized antibodies on the biosensor surface [1,2,3]. The immunoaffinity layer is essential for the specific recognition of target analytes, and is closely related to the sensitivity of the biosensor [4,5]. To improve the immunosensor sensitivity, the antigen-binding sites of antibodies should be maximally exposed during antibody immobilization [1]. Various methods have been utilized for antibody immobilization on transducers [6]. Among them, antibody orientation control has been proposed as a strategy for improving biosensor sensitivity [7,8]. Antibodies are asymmetric structures with small and localized antigen-binding sites, and randomly oriented antibodies expose less than 20% of the antigen-binding sites [9]. Therefore, orientation control can effectively improve the sensitivity of the immunoaffinity layer [10].

Affinity proteins, such as streptococcal protein G or staphylococcal protein A, have been used for antibody immobilization with orientation control [1,2,11]. Both bind to the Fc region of immunoglobulin G (IgG), and are therefore widely used for the purification of antibodies, as well as for orientation control [12]. For this reason, various studies have employed these affinity proteins in the immunoaffinity layer [13]. In previous studies, Z-domains were expressed on the outer membrane (OM) of *Escherichia coli* (*E. coli*), employing autodisplay technology and applied for immunoassaying and biosensing. Z-domains, an engineered 58-amino acids peptide from the B-domain of protein A, are also capable of IgG-binding [14]. Autodisplay, a surface display technology, expresses the target protein on the OM of gram-negative bacteria via fusion of autotransporters [15]. In autodisplay systems, the C-terminus of the target protein is expressed while being linked to the α-helical linker and the C-terminal of the linker is connected to the β-barrel, anchoring it to the OM [16]. IgG-binding Z-domains are automatically immobilized on OM with high density through autotransporters; thus, effective immobilization and orientation control can be realized by autodisplay technology. For these reasons, Z-domain-autodisplaying *E. coli* have been utilized in immunoassays and biosensors to improve sensitivity through orientation control [7,17].

In addition to the sensitivity of biosensor, reusability is highly advantageous in terms of the simplicity, reducing analysis time, saving materials resources and low experimental cost; therefore, various methods based on chemical, thermal and electrochemical treatments have been implemented to regenerate biosensor immunoaffinity layers [18]. In the case of immunosensors, harsh treatment such as thermal and electrochemical treatment is not suitable because of the denaturation of antibodies and other protein components in the immunoaffinity layer. Nevertheless, to realize the regenerable immunosensor, chemical immobilization of antibodies via chemical cross-linking is required to resist the regeneration processes, as the chemical cross-linking creates a strong bond between the target protein and solid support [1,19,20]. Crosslinkers covalently link proteins and impart additional properties according to their reactivity and spacing arm length [21,22]. Because the reactive ends of crosslinkers bind to specific functional groups of the target molecules, crosslinkers have been widely utilized to characterize biomolecule interactions, such as the relationship between adjacent proteins, ligand-receptor interactions, three-dimensional protein structures and molecular association in cell membranes [23,24]. Glutaraldehyde (GA) is one of the most frequently used crosslinkers for protein immobilization or fixation due to its reactivity with the amine group at room temperature and has been applied in various fields, such as histochemistry, cytochemistry, enzyme technology, sterilization, pharmaceutical chemistry and immunochemistry [25,26]. However, GA is toxic and produces toxic gases that irritate the eyes and skin, causing dyspnea [27]. In addition, because the shelf life of GA is shorter than one year, it must be freshly prepared [28]. Moreover, when GA links an antibody and protein, cross-linking is uncontrolled and may occur at various non-targeted sites; therefore, it is not ideal as a cross-linking agent for antibody immobilization [29]. *N*-hydroxysuccinimide (NHS) is an active ester widely used in peptide synthesis, amino group modification in proteins or cells and affinity chromatography, as it reacts with primary or secondary amines to form stable amide bonds [30,31]. For this reason, NHS ester can be replacement of GA used for antibody labeling, as well as an active group for cross-linking [29].

Previously, a regenerable immunoaffinity layer based on Z-domain-autodisplaying *E. col*i was developed and applied in surface plasmon resonance (SPR) biosensor fabrication [32]. The Oms with autodisplayed Z-domains were isolated and layered on the transducer of the SPR biosensor, and antibodies were immobilized with orientation control. Then, the sensor was treated with optimized acidic regeneration buffer and additive detergent for regeneration. The acidic regeneration buffer deformed the protein structure slightly, leading to antigen elution from the immunocomplex due to a decrease in the binding affinity. This regeneration strategy effectively improved the sensitivity as a result of orientation control, but the antibody immobilization step had to be repeated at every regeneration step. Antibodies were bound to Z-domains via hydrogen bonds, as was the case with antigen-antibody immunocomplexes; therefore, antibodies were also eluted from the Z-domains in the regeneration step.

In this study, a regenerable immunoaffinity layer with both chemically immobilized and orientation-controlled antibodies was generated based on autodisplay technology and crosslinker treatment. The Oms of Z-domain-autodisplaying *E. coli* were isolated and layered on a solid support, and antibodies were immobilized with orientation control to form an immunoaffinity layer. Then, a disuccinimidyl crosslinker was used to fix the immobilized antibodies to autodisplayed Z-domains via covalent bonding. This covalently immobilized orientation-controlled antibody layer was not eluted during the regeneration process. Regenerable assaying and biosensing based on the fabricated immunoaffinity layer were conducted, and their applicability for medical diagnosis was confirmed.

## 2. Results and Discussion

### 2.1. Optimization of Chemical Immobilizing Conditions

In general, chemical immobilization of antibodies is achieved by sequential treatment with crosslinkers and antibodies. Surface activation via crosslinker treatment can offer effective and dense immobilization sites, but cannot provide antibody orientation control. In this study, antibodies were chemically fixed following orientation control. Although antibody orientation control would likely be maintained, there was a possibility of suppressed antigen binding affinity due to the binding of the crosslinker to the antigen binding sites. To determine the optimal crosslinker for the chemical immobilization of antibodies without decreasing the antigen-binding activity, homobifunctional NHS esters with various spacer arms, including disuccinimidyl glutarate (DSG), disuccinimidyl suberate (DSS) and poly (ethylene glycol)-ylated bis (sulfosuccinimidyl)suberate (BS(PEG)_5_), were tested. The spacer arms of DSG, DSS and BS(PEG)_5_ are known to be 7.7, 11.4 and 21.7 Å in length, respectively. In addition, the ether bond in BS(PEG)_5_ is expected to provide flexibility. As mentioned above, because GA is one of the most frequently used crosslinkers, it was used as a comparison control.

**Figure 1:**
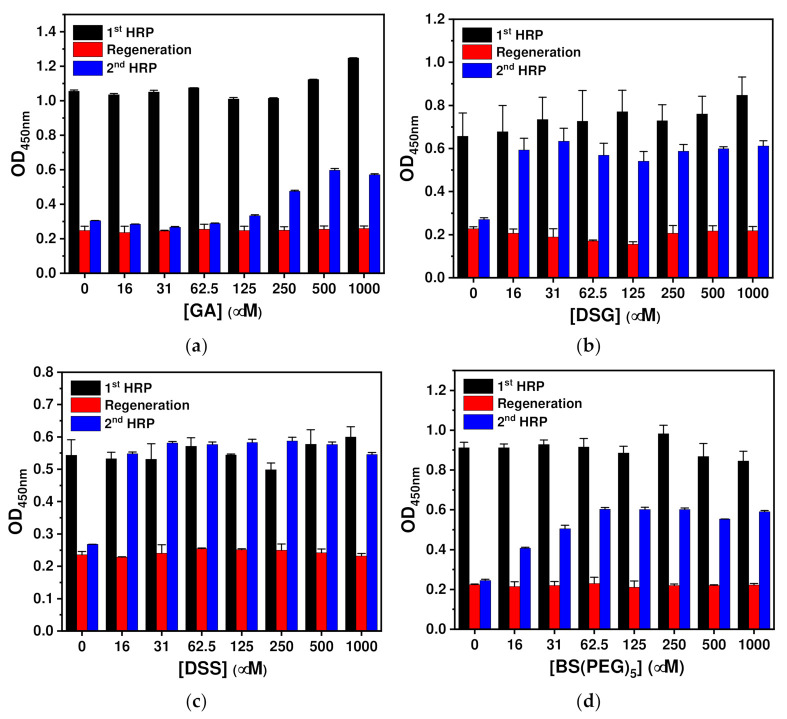
Determination of optimal crosslinker concentrations. OD values obtained during the regenerable HRP immunoassay after chemical immobilization with varying concentrations of (**a**) GA and those obtained using (**b**) DSG, (**c**) DSS and (**d**) BS(PEG)_5_. For the regenerable test, HRP treatment was performed before and after the regeneration step, and OD values were obtained after the HRP-TMB reaction in the presence of peroxide from each regenerable assay step. The error bar means a standard deviation.

To optimize the crosslinker concentration, a regenerable test was performed using Z-domain-autodisplaying *E. coli* cells. Horseradish peroxidase (HRP) was selected as a model analyte, as it could be quantified directly without the use of detection antibodies via reaction with 3,3′,5,5′-tetramethylbenzidine (TMB) in the presence of peroxide. Z-domains with Fc region binding activity were autodisplayed on the OM of *E. coli*, and these cells were used as a solid support for the immunoassay (Figure 1). After immobilization of the capture antibodies with orientation control on *E. coli* cells, GA, DSG, DSS and BS(PEG)_5_ at varying concentrations (0–1 mM) were used for chemical immobilization. After the washing step, 10 ng/mL of HRP was added and the optical density (OD) resulting from the TMB reaction was measured at a wavelength of 450 nm. Then, regeneration was performed by treatment with a detergent-containing acidic buffer. After regeneration, the same concentration of HRP was added, and bound HRP was quantified based on the TMB reaction results.

As shown in Figure 1a, orientation-controlled antibodies bound to the autodisplayed Z-domains were chemically immobilized using GA. Relative OD values were calculated using those of the first HRP treatment as a reference and the OD values of the regeneration step were <25% relative to those of the first HRP treatment in the entire range. This indicated that the regeneration step efficiently eluted the analyte from the capture antibodies. HRP was then added to the regenerated *E. coli* cells and OD values similar to those of the regeneration step were obtained at concentrations of ≤125 μM. At concentrations of >250 μM, the relative OD values increased until a relative OD value of 53% at 500 μM. This suggests that GA treatment did not chemically immobilize anti-HRP antibodies – hence, they were eluted together with the antigen and the regeneration buffer.

DSG was tested for the chemical fixation of orientation-controlled antibodies bound to autodisplayed Z-domains (Figure 1b). As shown in Figure 1b, the second HRP treatment gave rise to higher relative OD values ranging between 71% (125 μM) and 89% (31 μM) compared to those of the GA treatment, but all of the OD values were <100%. As shown in Figure 1c, DSS was tested for the chemical fixation of orientation-controlled antibodies. The second HRP treatment resulted in relative OD values between 93% (1 mM) and 120% (250 μM), being higher than those of the GA and DSG treatments. As the relative OD values were close to 100%, it can be concluded that DSS treatment was more effective for fixing anti-HRP antibodies than the other tested linker treatments. Lastly, BS(PEG)_5_ was tested for the chemical fixation of orientation-controlled antibodies. As shown in Figure 1d, the relative OD values resulting from the second HRP treatment increased from 45% (16 μM) to 70% (1 mM), showing intermediate results between those of GA and DSG treatment. From the regenerable test, optimum concentrations of cross-linkers were selected as the concentration showing the highest relative OD value of the second HRP treatment, and were determined as 500, 31, 500 and 125 μM for GA, DSG, DSS and BS(PEG)_5_, respectively. Those relative OD values were 53%, 89%, 102% and 68% for GA, DSG, DSS and BS(PEG)_5_, respectively; thus, DSS showed the highest efficiency in regenerable test with a single regeneration step.

**Figure 2:**
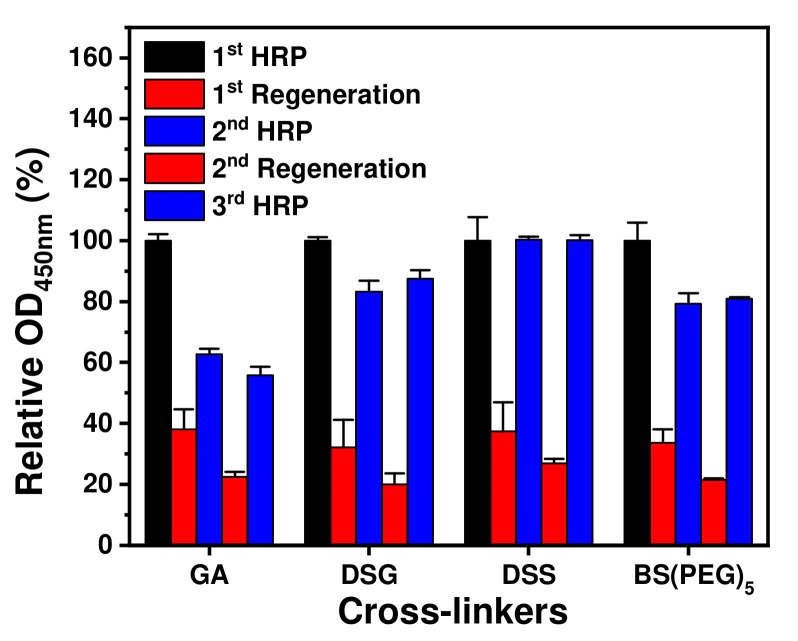
Crosslinker optimization. GA, DSG, DSS and BS(PEG)_5_ were treated after binding of capture antibodies to autodisplayed Z-domains at concentrations of 500, 31, 500 and 125 μM, respectively. Regenerable HRP immunoassays were repeated three times with two regeneration steps.

From the above results, optimum concentrations of GA, DSG, DSS and BS(PEG)_5_ were determined as 500, 31, 500 and 125 μM, respectively. Using these optimum concentrations, capture antibodies were chemically fixed after binding with Z-domains autodisplayed on *E. coli* Oms. As shown in Figure 2, the HRP immunoassay was repeated three times with two regeneration steps. Relative OD values at 450 nm were calculated using those of the first HRP treatment as a reference. Regardless of the type of crosslinker, low relative OD values of <40% were obtained after all of the regeneration steps. This indicated that the regeneration buffer effectively eluted antibody-bound analytes. In the GA treatment, the relative OD values decreased by 62% and 56% as the number of regeneration cycles increased. These results indicate that GA treatment cannot covalently immobilize the antibodies, and therefore the chemically unfixed antibodies were eluted during the regeneration steps. In the case of DSG treatment, the relative OD values of the second and third HRP steps were 83% and 87%, respectively. BS(PEG)_5_ treatment gave rise to lower values than DSG treatment after the regeneration steps. Relative OD values for the BS(PEG)_5_ treatment after the second and third HRP steps were 79% and 81%, respectively. Although the values were higher than those arising from GA treatment, both DSG and BS(PEG)_5_ treatments were not suitable for efficient antibody immobilization through covalent bonds as the relative OD values obtained for the HRP treatment after regeneration were significantly lower than 100%. For DSS treatment, the relative OD values obtained after the second and third HRP steps were close to 101% and 100%, respectively. This indicated that DSS treatment did not affect the antigen-binding activity of the antibody and effectively fixed the capture antibodies through covalent bonding. Based on these results, DSS treatment at a concentration of 500 μM was selected as the optimum condition for covalent immobilization of antibodies bound to autodisplayed Z-domains with orientation control.

### 2.2. Regenerable Immunoassay Based on Covalently Immobilized Antibodies with Orientation Control

Regenerable immunoassays based on Z-domain-autodisplaying *E. coli* cells and their OMs bearing covalently immobilized orientation-controlled antibodies were performed under optimal conditions. For the *E. coli*-cell-based regenerable HRP immunoassay, Z-domain- autodisplaying *E. coli* cells were utilized as the solid support with antibody orientation control. After anti-HRP antibody binding to the Z-domains, the DSS crosslinker (500 μM) was added for chemical immobilization. Then, the analyte, 20 ng/mL of HRP, was introduced and quantified based on its reaction with TMB in the presence of peroxide. For regeneration following the HRP immunoassay, analytes were eluted by treatment with 2% tween-20 in 100 mM glycine buffer at pH 2.5. The HRP immunoassay was repeated four times, with three regeneration steps.

**Figure 3:**
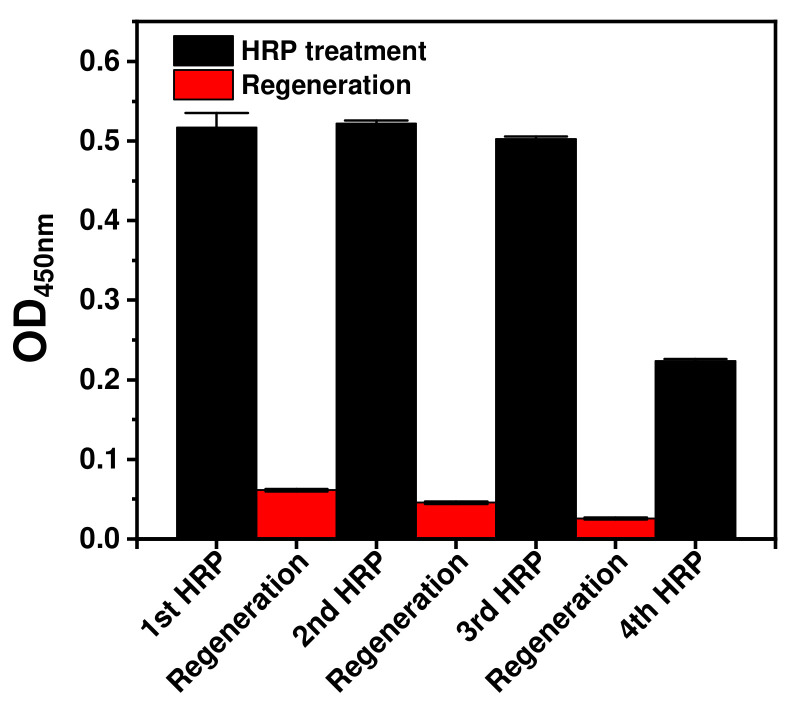
Regenerable immunoassays using covalently immobilized orientation-controlled antibodies based on Z-domain- autodisplaying *E. coli* cells as solid support.

As shown in Figure 3, the OD values of the first, second, third and fourth HRP immunoassays were 0.52, 0.52, 0.50 and 0.22, respectively. The OD values of the second and third immunoassays were nearly 100%, relative to that of the first HRP immunoassay. Meanwhile, the OD value of the fourth HRP immunoassay decreased by 43% compared to that of the first. The *p*-value of the Student’s *t*-test between the fourth and first HRP immunoassay OD values was <0.0001. The *p*-value of <0.05 indicated that the fourth HRP immunoassay differed significantly from the previous immunoassays. In the case of immunoassays based on *E. coli* cells, the assay signal was validated to be considerably close to 100% until the third HRP treatment. The decrease in the OD values after the fourth HRP treatment may arise from repeated centrifugation. *E. coli* cells require several centrifugation steps for each buffer change and washing step, and repeated spin-down and resuspension processes result in the loss of *E. coli* cells. As a result of the decrease in the solid support, the number of *E. coli* cells in the immunoassay decreased after the third regeneration step. The OD values of the regeneration steps were 0.06, 0.05 and 0.03, respectively, from the first to the third regeneration step. Compared with the OD value of the first HRP immunoassay, the OD values of the first to the third regeneration steps were 12%, 9% and 5% and all the *p*-values between each regeneration step and the first HRP immunoassay were <0.0001. These results indicate that the signals decreased significantly after each regeneration step. Then, when HRP was treated the signal nearly 100% was recovered as mentioned above. From these data, it was concluded that treatment of autodisplayed Z-domain-bound orientation-controlled antibodies with DSS can effectively fix them through covalent bonds, and *E. coli* cells with covalently immobilized orientation-controlled capture antibodies are a suitable solid support for regenerable immunoassays.

To avoid signal decrease due to cell loss and biosensor use, the OMs of Z-domain-autodisplaying *E. coli* cells were isolated as liposomes by sequential treatment with lysozyme and the Triton X-100 detergent, and isolated OM particles were layered on a 96-well microplate, a two-dimensional substrate, via hydrophobic interactions [2,33]. After coating with the OMs, a covalently immobilized and orientation-controlled immunoaffinity layer was formed by sequential treatment with capture antibodies and DSS. As this immunoaffinity layer on the surface of the microplate did not require centrifugation steps during buffer change and washing, it was expected to prevent signal lowering associated with regenerable immunoassay processes. After formation of the immunoaffinity layer, 20 ng/mL of HRP was introduced, followed by TMB for quantification. Similar to the *E. coli*-cell-based immunoassay, regeneration of the OM-based immunoassay was performed, and immunoassays were repeated five times with four regeneration steps.

**Figure 4:**
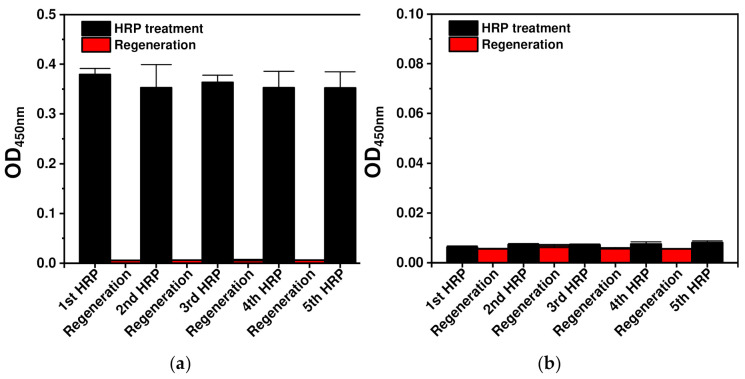
Regenerable OM-based immunoassays comprising covalently immobilized orientation-controlled antibodies using (**a**) Z-domain-autodisplaying *E. coli* and (**b**) intact *E. coli*. Anti-HRP antibodies were immobilized on the Z-domain-autodisplaying OM layer and chemically fixed by treatment of DSS.

The results of the OM-based HRP regenerable immunoassay are shown in Figure 4a. The OD values obtained from the first to the fifth HRP immunoassay were 0.38, 0.35, 0.36, 0.35 and 0.35, respectively. In comparison with the first HRP immunoassay, the relative OD values from the second to fifth immunoassays were 93%, 96%, 93% and 93%, respectively. This means that all of the HRP immunoassays gave rise to stable signals and the regenerable immunoassay was validated with <10% deviation until the fifth HRP treatment. The OD values of the regeneration steps were all <0.01, and the relative OD and *p*-values of the regeneration steps in comparison with those of the first HRP immunoassay were all <2% and 0.001, respectively. These results indicated that the signals statistically significantly decreased by <2% after the regeneration steps. Compared to the *E. coli-*cell-based immunoassay (Figure 3), the OM-based immunoassay (Figure 4) gave rise to especially low relative OD values after the regeneration steps. This is likely due to *E. coli* enzymes. *E. coli* cells contain various enzymes, including peroxidase, in the periplasm and cytosol, which may be released from lysed cells during repeated centrifugation steps conducted during *E. coli-*based immunoassays. In the case of OM-based immunoassays, only high-purity OMs, from which enzymes in the periplasm and cytosol were removed, are isolated and layered to form the immunoaffinity layer [33]. Thus, OM-based immunoassays can detect especially low background signals. These data verify that treatment using DSS under optimal conditions efficiently fixes autodisplayed Z-domain-bound antibodies via covalent bonding, and provides resistance to elution by the acidic regeneration buffer.

To test the effect of autodisplayed Z-domains, the OMs of intact *E. coli* cells without an autodisplaying vector were used as a comparison group. As shown in Figure 4b, the OD and *p*-values obtained for all the HRP immunoassays and regeneration steps were < 0.01 and 0.001, respectively. These results confirm that autodisplayed Z-domains efficiently bind antibodies and prevent non-specific binding of *E. coli* in OM-based immunoassays during regeneration. From these data, it was confirmed that treatment with DSS provides robust covalent bonding between orientation-controlled capture antibodies and the Z-domain-autodisplaying OM layer, creating an efficient immunoaffinity layer suitable for regenerable immunoassays. In addition, the analyte binding capacity of the capture antibodies was maintained after treatment with both the crosslinker and regeneration buffer.

### 2.3. Application of Covalently Immobilized Regenerable Immunoaffinity Layer with Orientation Control in an SPR Biosensor

The developed immunoaffinity OM-based layer with covalently immobilized orientation-controlled antibodies was applied to obtain an SPR biosensor. The transducer surface in SPR biosensors generally consists of metals such as silver or gold, and in this study, an SPR biosensor with a bare gold surface was utilized. The gold surface is hydrophobic, and the isolated OM particles are bilayers comprising hydrophilic lipopolysaccharides in the outer layer and hydrophobic lipoprotein-containing inner layer. Similar to layering on a microplate, isolated OM particles from Z-domain-autodisplaying *E. coli* cells formed a layer on the gold surface of the SPR biosensor through hydrophobic interactions [2]. SPR biosensors are frequently applied to quantify C-reactive protein (CRP), widely regarded as an acute-phase inflammation biomarker for the diagnosis of various diseases, including inflammatory diseases and obesity [34]. Following the formation of the OM layer on the gold surface of the SPR biosensor, it was treated with capture antibodies for 1 h to form an orientation-controlled antibody layer on the autodisplayed Z-domains on the OM. Subsequently, DSS was added to covalently bond the antibodies to the autodisplayed Z-domains. After fixation with the crosslinker, CRP was introduced at a concentration of 100 ng/mL. After CRP quantification, the analyte bound on the immunoaffinity layer was eluted by treatment with an acidic regeneration buffer. Following the regeneration step, CRP was added again for assessment. CRP measurements were repeated five times with five regeneration steps.

**Figure 5:**
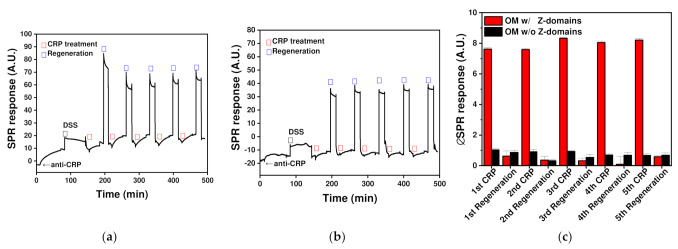
SPR responses using a covalently immobilized regenerable immunoaffinity layer bearing orientation-controlled antibodies based on: (**a**) OMs with autodisplayed Z-domains; and (**b**) OMs without an autodisplaying vector. (**c**) Relative SPR responses in comparison with the SPR response of the immunoaffinity layer.

The SPR response was monitored, as shown in Figure 5a. After treatment with anti-CRP antibodies, the SPR response increased by 11.85 A.U. and 3.28 A.U., relative to the SPR response increase after DSS treatment. This supports the hypothesis that antibodies bind to the OM layer and form covalent bonds with the autodisplayed Z-domains as a result of DSS treatment. Following the formation of the immunoaffinity layer, regenerable biosensing was performed by repeating the CRP treatment and regeneration steps. In comparison with the SPR response of the immunoaffinity layer, the signal increments from the first to fifth CRP treatment were calculated to be 7.62, 7.59, 8.33, 8.05 and 8.21 A.U., and the relative signal responses from the second to fifth CRP treatment were 100%, 109%, 106% and 108%, respectively, in comparison with that of the first CRP treatment (Figure 5c). This indicates that the signals from five regenerable biosensing cycles were stable with <10% deviation. In addition, the coefficient of variation indicative of SPR biosensing repeatability was calculated to be 4.27%. This result indicates that regeneration using the demonstrated immunoaffinity layer exhibited good repeatability. In the case of the regeneration steps, a high SPR response was monitored during regeneration buffer treatment. This was because the composition and concentration of the regeneration buffer differed from those of the other PBS-based solutions. All SPR responses were measured using the same PBS solution after the washing step to avoid buffer composition or concentration effects. In comparison with the immunoaffinity layer SPR response, signals from the first to the fifth regeneration steps were calculated to be 0.63, 0.36, 0.32, 0.09 and 0.58 A.U., respectively. All signals differed by <8% in comparison with the signal from the first CRP treatment. This confirms that the immunoaffinity layer on the SPR biosensor was successfully regenerated. These data support that the immunoaffinity layer, comprising covalently immobilized orientation-controlled antibodies, was formed on the SPR biosensor comprising autodisplayed Z-domains and the fabricated immunoaffinity layer was reusable after regeneration.

For the comparison group, the OM layer derived from intact *E. coli* cells without an autodisplaying vector was utilized and the SPR response is shown in Figure 5b. After the formation of the OM layer, the SPR responses changed by 0.12 and 2.78 A.U., following sequential anti-CRP and DSS treatment, respectively. Antibody treatment induced a signal change of <1.5%, which was significantly lower than observed for the OM layer of autodisplaying-vector-bearing *E. coli*, as there was no autodisplayed Z-domain to bind with the antibodies in the former. Although there were no immobilized antibodies, DSS could fix other membrane proteins in the OM layer, and thus the SPR response changed by 2.78 A.U. After treatment with DSS, the magnitudes of the SPR response increments from the first to the fifth CRP treatment were 1.02, 0.91, 0.93, 0.68 and 0.66 A.U. and those from the first to the fifth regeneration step were 0.86, 0.32, 0.53, 0.67 and 0.68 A.U., respectively. All signals were less than 15% lower in magnitude in comparison with those arising after CRP treatment using an affinity layer with autodisplayed Z-domains. This indicated that the developed OM layer applied to the SPR biosensor efficiently prevented non-specific binding of antibodies or analytes and autodisplayed Z-domains containing the OM layer were suitable for the formation of an effective immunoaffinity layer. These results confirmed that a regenerable immunoaffinity layer with covalently immobilized orientation-controlled antibodies was successfully formed on the SPR biosensor surface, and it could be successfully regenerated five times.

In this study, the regenerable immunoaffinity layer comprising covalently immobilized orientation-controlled antibodies was developed and applied to the immunoassays and SPR biosensor. The ability to conduct five regeneration cycles and five CRP measurements on a single SPR chip is both time- and cost-efficient, as the immunoaffinity layer is generated only once. Controlling the antibody orientation via autodisplay technology improved the sensitivity of the SPR biosensor [10]. In addition to antibody orientation control, covalent immobilization employing a crosslinker increases the stability of the immunoaffinity layer; therefore, cost and time efficient sensitive and regenerable biosensing was realized.

## 3. Materials and Methods

### 3.1. Reagent

GA, DSS, DSG, BS(PEG)_5_, HRP, CRP, tween-20, Triton X-100, isopropyl β-D-1-thiogalactopyranoside (IPTG), ethylenediaminetetraacetic acid (EDTA), glycine and hydrochloric acid were purchased from Merck (Darmstadt, Germany). Rabbit polyclonal antibodies against HRP, rabbit polyclonal antibodies against CRP and fluorescein-conjugated goat polyclonal antibodies against CRP were obtained from Abcam (Cambridge, UK). Phosphate-buffered saline (PBS) was purchased from LPS solution (Daejeon, Korea) and used as an antibody-dissolving buffer. In addition, 96-well microplates were obtained from SPL Life Sciences (Pocheon, Korea). The TMB substrate kit was purchased from Thermo Fisher Scientific (Waltham, MA, USA).

### 3.2. Cultivation of Autodisplaying E. coli Cells and OM Isolation

A Z-domain autodisplaying vector was constructed by cloning the Z-domain, as described in previous studies [32]. Z-domain autodisplaying vectors were transformed into *E. coli* cells (BL21(DE3)) via electroporation. The transfected *E. coli* cells were grown overnight. After overnight cultivation, *E. coli* cells were induced with IPTG. After induction, *E. coli* cells were harvested and washed three times by centrifugation. The OMs of Z-domain-autodisplaying *E. coli* cells were isolated according to previous studies [33]. In brief, the rigid peptidoglycan layer of *E. coli* cell walls was hydrolyzed by lysozyme treatment, resulting in the formation of spheroplasts. To assist lysozyme penetration into the periplasm, sucrose and EDTA were added to enhance osmosis and control the surface charge. Following spheroplast formation, the OMs of *E. coli* cells were isolated as a liposome by the addition of Tris-HCl (pH 8) containing the detergent, Triton X-100. After the purification of OM particles by sequential centrifugation, the isolated OMs were dissolved in PBS.

### 3.3. Regenerable Test of Covalently Immobilized Immunoaffinity Layer with Orientation Control

For the *E. coli*-cell-based regenerable assay, Z-domain-autodisplaying *E. coli* cells were adjusted to an OD_600_ value of 1.0. Next, anti-HRP antibodies at a concentration of 1.0 μg/mL were added to the cells. Following incubation for 1 h, the *E. coli* cells were washed three times by sequential centrifugations. Then, 100 μL of the crosslinker was added for covalent fixation of the antibodies. After washing, 10 ng/mL HRP was added and the cells were incubated for 1 h. Following the washing steps, 100 μL of regeneration buffer, 2% tween-20 containing 0.1 M glycine buffer at pH 2.5, was added to selectively elute the analyte from the antibody-immobilized *E. coli* cells. After regeneration, the *E. coli* cells were washed three times and resuspended in 100 μL of PBS. These regenerated *E. coli* cells with covalently immobilized antibodies were used as a new solid support for immunoassaying. The chromogenic reaction with TMB was used to quantify bound HRP. After quenching with 2 M sulfuric acid, the OD value was measured at 450 nm using a microplate reader (Molecular Devices, San Jose, CA, USA).

For the OM-based regenerable assay, isolated OMs were added to a 96-well microplate and incubated for 2 h to form the OM layer on the surface of the microplate. After OM layer formation, the plate was washed three times and treated with capture antibodies at a concentration of 1.0 μg/mL for 1 h. Subsequently, the microplate was washed and treated with a crosslinker for fixation. Following the washing steps, the layer was treated with analytes for 1 h. After immunocomplex formation, regeneration buffer was added for 2 min, and the eluted antibodies and antigens were washed immediately. The regenerated OM layer with covalently immobilized antibodies was used as a new solid support for immunoassaying and biosensing. For quantification, the TMB solution was added and the reaction was quenched by the addition of sulfuric acid. All assays were performed in triplicate.

### 3.4. SPR Biosensor Measurement

A commercial SPR biosensor system sourced from ICLUEBIO Co., Ltd. (Seongnam, Korea) was used in this study. For OM-based regenerable biosensing, an unmodified bare gold chip was used. For immunoaffinity layer formation, the surface of the gold chip was treated with the isolated OM solution (300 g/mL) for 2 h. The concentration of OM solution was measured using BCA Protein Assay Kit (Thermo Fisher Scientific, Waltham, MA, US). The remaining OM isolated particles were washed with phosphate-buffered saline (PBS). After OM layer formation, anti-CRP antibodies (1 μg/mL in PBS) were bound to autodisplayed Z-domains on the OM layer. After washing, the crosslinker was used to covalently fix the antibodies. The sensor was then treated with CRP samples for 30 min. This was followed by treatment with regenerable solution for 2 min to regenerate the OM immunoaffinity layer with autodisplayed Z-domains. Following the regeneration step, CRP biosensing was repeated four times using the regenerated OM layer on the gold chip of the SPR biosensor. Captured CRP was quantified by subtracting the SPR signal after CRP treatment from the SPR signal before CRP treatment. The regeneration rate was calculated by comparing the SPR signal after OM layer formation with the SPR signal following regeneration. All treatments steps were measured in a static condition, and washing steps were carried out in a flow mode (1 mL/min).

## 4. Conclusions

A regenerable immunoaffinity layer was developed employing a crosslinker and Z-domain autodisplaying technology to covalently immobilize antibodies with orientation control. For antibody orientation control, Fc region-binding Z-domains were autodisplayed on the OMs of *E. coli* cells and three types of disuccinimidyl crosslinkers, namely DSG, DSS and BS(PEG)_5_, and widely used GA were tested for chemical fixation. DSS at a concentration of 500 μM was determined to be optimal for the covalent fixation of antibodies bound to autodisplayed Z-domains, and this process was confirmed not to affect the antigen-binding activity of antibodies. Regenerable HRP immunoassays based on *E. coli* cell OMs were performed. After the formation of an immunoaffinity layer comprising covalently bonded orientation-controlled antibodies, HRP treatment was conducted and repeated following the regeneration steps. In the case of immunoassays based on *E. coli* cells, the results confirmed that three sequential regenerable immunoassays were feasible with <2% variation. To avoid signal decrease due to centrifugation, the OMs of Z-domain-autodisplaying *E. coli* were isolated and an OM-based immunoassay was demonstrated. The results confirmed that five sequential regenerable immunoassays were feasible with <10% variation. In addition, the OM layer was confirmed to effectively prevent non-specific binding as a result of antibody orientation control and the retention of antigen-binding properties. This regenerable OM layer with covalently immobilized capture antibodies was applied as an immunoaffinity layer of an SPR biosensor and tested using CRP. According to SPR measurements, immunoaffinity layer formation was confirmed and validated, following five repeated measurements after regeneration. The results indicated that the SPR signals obtained from five regenerable biosensing cycles were stable with <10% deviation, and the coefficient of variation for SPR biosensing was calculated to be 4.27%, indicative of good repeatability. From these data, it was concluded that the fabricated immunoaffinity layer is suitable for regenerable immunoassay and biosensor applications. The regenerable immunoassays and SPR biosensor application without the treatment of a cross-linker was performed in a previous study [32]. In that study, capture antibodies immobilized on autodisplayed Z-domains were eluted by the treatment of the regeneration buffer, so the antibody addition was essential for the reuse of immunoaffinity layer. Meanwhile, in this study, antigens bound to the immunoaffinity layer were selectively eluted by treatment with acidic regeneration buffer and the OM layer with covalently immobilized orientation-controlled antibodies was readily reconstructed. Thus, the chemical fixation of antibodies by the crosslinker treatment in this study is the advance technology for the construction of the reusable immunoaffinity layer. Application of the developed regenerable immunoaffinity layer to various immunosensors may improve sensitivity as a result of antibody orientation control, and reduce the total cost of medical diagnosis as the transducer and immunoaffinity layer are reusable.
